# Kinetic Spectrophotometric Determination of Fluvastatin in Pharmaceutical Preparations

**Published:** 2010-03

**Authors:** Safwan Ashour, Mahmoud Bahbouh, Mouhammed Khateeb

**Affiliations:** 1 *Department of Chemistry, Faculty of Sciences, University of Aleppo, Aleppo, Syria;*; 2 *Department of Chemistry, Faculty of Sciences, University of Al-Baath, Homs, Syria*

**Keywords:** fluvastatin, kinetic spectrophotometry, 4-chloro-7-nitrobenzofurazan (NBD-Cl), pharmaceutical dosage forms, method validation

## Abstract

Simple, accurate and reliable kinetic spectrophotometric method for the determination of fluvastatin sodium (FVS) in pure form and pharmaceutical formulations has been described. The method is based on the formation of colored product between FVS and 4-chloro-7-nitrobenzofurazan (NBD-Cl) in acetone medium at 55 ± 2ºC. The reaction is followed spectrophotometrically by measuring the increase in absorbance at 462 nm as a function of time. The rate data and fixed time methods were adopted for constructing the calibration curves. The linearity ranges were found to be 15.0–50.0 and 10.0–90.0 μg mL^−1^ for rate data and fixed time methods, respectively. The limit of detection for rate data and fixed time methods is 0.017 and 0.134 μg mL^−1^, respectively. The proposed methods have been successfully applied to the determination of fluvastatin sodium in pharmaceutical dosage forms with no interference from the excipients. Statistical comparison of the results shows that there is no significant difference between the proposed and official methods.

## INTRODUCTION

Fluvastatin sodium (FVS) is (3R,5S,6E)-rel-7-[3-(4-fluorophenyl)-1-(1-methylethyl)-1H-indol-2-yl]-3,5-dihyroxy-6-heptenoicacid, monosodium salt, is a competitive inhibitor of HMG-CoA reductase, which is responsible for the conversion of 3-hydroxy-3-methylglutaryl-coenzyme A (HMG-CoA) to mevalonate, a precursor of sterols including cholesterol. It is used to reduce triglycerides, LDL-cholesterol, apoliporotein B and to increase HDL-cholesterol, in the treatment of hyperlipidaemias including hypercholesterolaemias and combined hyperlipidaemia. FVS is metabolized in the liver, primarily via hydroxylation of the indole ring at the 5 and 6-positions. N-dealkylation and beta-oxidation of the side-chain also occurs. The hydroxyl metabolites have some pharmacological activity, but do not circulate in the blood. FVS has two optical enantiomers, an active 3R, 5S and an inactive 3S, 5R form. FVS is 98% bound to plasma proteins (1–3).

Literature survey reveals that FVS is official in U.S.P. (4). Several electroanalytical methods are available for the determination of the latter compound by differential plus voltammetry (5), square-wave adsorptive-stripping voltammetry (SWAdSV) (6) in pharmaceutical preparations, cyclic voltammetry (7) and variety of voltammetric techniques (8) in pharmaceutical dosage forms and biological fluids. FVS has been determined by capillary electrophoresis (CE) (9), first derivative spectrophotometry (10), High performance liquid chromatography (HPLC) with fluorescence detector (11–16) and ultraviolet detector (17, 18), Liquid chromatography/negative ion electrospray tandem mass spectrometry (19), Liquid chromatography/electrospray mass spectrometry (20), and gas chromatography/negative ion chemical ionization mass spectrometry (21) in pharmaceutical formulations and biological samples.

No kinetic spectrophotometric methods have been reported in the literature for the assay of FVS. Some specific advantages that the kinetic methods possess are as follows (22): simple and fast methods because some experimental steps such as filtration, extraction, etc. are avoided prior to absorbance measurements, high selectivity since they involve the measurement of the absorbance as a function of reaction time instead of measuring the concrete absorbance value, other active compounds present in the commercial dosage forms may not interfere if they are resisting the chemical reaction conditions established for the proposed kinetic method and colored and/or turbid sample background may possibly not interfere with the determination process.

This paper describes a simple and sensitive kinetic spectrophotometric method for the determination of FVS in bulk and drug formulations. The method is based on the reaction between FVS and 4-chloro-7-nitrobenzofurazan (NBD-Cl) in acetone medium resulting in the formation of yellow color, which absorbs maximally at 462 nm. The absorbance increases with time and therefore, two calibration procedures ie, rate data and fixed time methods are adopted for the determination of FVS in commercial dosage forms.

## EXPERIMENTAL

### Apparatus

A Jasco V-530 UV-VIS spectrophotometer (Japan) with 1 cm quartz cells was used for all absorbance measurements under the following operating conditions: scan speed medium (400nm/min), scan range 375–550nm and slit width 2nm. Spectra were automatically obtained by Jasco system software. pH measurements were made with Consort C 830 (Belgium) with combined glass pH electrode. A water bath shaker (Grant instruments, Cambridge Ltd, England) was used to control the heating temperature for color development.

### Materials and Reagents

Fluvastatin sodium (C_24_H_25_FNO_4_Na, 433.46g mole^−1^) was supplied by ALPHARM Chemical Co (China). Its purity was found to be 99.2% according to the compendial method. 4-chloro-7-nitrobenzofurazan (NBD-Cl) was purchase from Aldrich company. All other chemicals and reagents used were of analytical grade and all solutions were prepared with double distilled water.

### Formulations

Almastatin capsules supplied by Alma company (Homs, Syria), each capsule was labeled to contains fluvastatin sodium 20 or 40 mg and fluvastatin capsules supplied by Kimi (Aleppo, Syria), each capsule was labeled to contain fluvastatin sodium 20 or 40 mg.

### Solutions

Standard stock solution of fluvastatin sodium in concentration of 0.5 mg mL^−1^ was prepared in 100mL volumetric flask by dissolving required amount of fluvastatin sodium with 3mL methanol, the volume was then diluted to the mark with acetone. The working standard solutions were freshly prepared by suitable dilution of the stock solution with acetone. NBD-Cl 0.2% solution was freshly prepared with acetone.

### General procedures

**Rate data method.** Aliquots of standard FVS solution (0.30–1.00 mL, 0.5 mg mL^–1^) were transferred into a series of 10 mL calibrated volumetric flasks. Then 0.75 mL of NBD-Cl solution was added and the volume was made up to the mark with acetone, mixed well and heated in the water bath at 55 ± 2°C. After mixing, the contents of each flask were immediately transferred to the spectrophotometric cell and the increase in absorbance was recorded at 462 nm as a function of time between 5–40 min against reagent blank treated similarly. The rate of the reaction (ν) at different concentrations was obtained from the slope of the tangent to the absorbance-time curve. The calibration curve was constructed by plotting the logarithm of the reaction rate (log ν) *vs* the logarithm of the molar concentration of the FVS (log C). The amount of the drug was obtained either from the calibration graphs or the regression equation.

**Fixed time method.** Aliquots of standard FVS solution (0.20–1.80 mL, 0.5 mg mL^−1^) were transferred into a series of 10 mL calibrated volumetric flasks. Then 0.75 mL of NBD-Cl solution was added and the volume was made up to the mark with acetone, mixed well and heated on a water bath at 55 ± 2°C for 20 min. After mixing, the contents of each flask were immediately transferred to the spectrophotometric cell and the absorbance was recorded at 462 nm against reagent blank treated similarly. The calibration curve was constructed by plotting the absorbance against the final concentration of the drug. The amount of the drug in each sample was computed either from calibration curve or regression equation.

### Procedures for formulations

The entire content of twenty capsules containing FVS were weighed and mixed well. Amount of the powder equivalent to 25 mg of FVS was dissolved in a 25 mL of methanol and mixed for about 5 min. and then filtered through Whatman filter paper number 40. The methanol was evaporated to about 1.5 mL. The remaining portion of the solution was diluted in a 50 mL volumetric flask to the volume with acetone to achieve a concentration of 0.5 mg mL^−1^. The general procedures were then followed in the concentration ranges mentioned above.

## RESULTS AND DISCUSSION

### Absorption spectra

**Figure 1:**
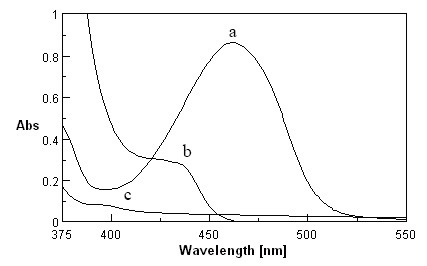
Absorption spectra of (a) 50 µg mL^−1^ FVS + 0.75 mL NBD-Cl 0.2% against reagent blank; (b) reagent blank (NBD-Cl 0.2%) against acetone; (c) 50 µg mL^−1^ of FVS against acetone.

4-chloro-7-nitrobenzofurazan (NBD-Cl), as an electroactive halide reagent, was first introduced as an analytical reagent for the determination of some amines and amino acids (23). Several pharmaceutical compounds have been determined through this approach, such as, tramadol (24) and isoxsuprine (25). In the present study, FVS (alkylamine such as tramadol and isoxsuprine) was found to react with NBD-Cl in acetone medium at 55 ± 2°C producing a yellow color with maximum absorbance at 462nm (Fig. 1).

### Optimization of reaction conditions

The optimum conditions for the development of method were established by varying the parameters one at a time and keeping the others fixed and observing the effect produced on the absorbance of the colored product. In order to establish experimental conditions, the effect of various parameters such as solvents, temperature, concentration of NBD-Cl and time of heating were studies.

When using acidic, neutral or basic buffer media such as britton buffer and borate buffer, reagent forms an orange yellow color. This will decrease the absorbance of the sample solution when using it as blank. Several organic solvents i.e. methanol, ethanol and acetone were investigated. Acetone was found to be the best solvent for formation of colored product.

**Figure 2:**
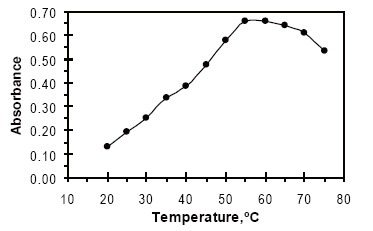
Effect of temperature on the formation of colored product FVS-NBD-Cl, [FVS] = 50 µg mL^−1^ + 1mL NBD-Cl 0.2% for 10 min.

The effect of temperature on the reaction was studied in the range of 20–75°C. 55°C was found to be optimal for maximum color development (Fig. 2).

**Figure 3:**
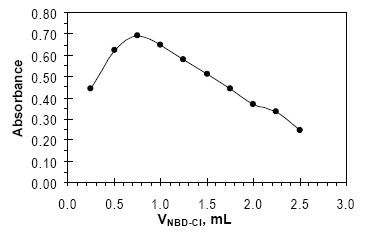
Effect of concentration of NBD-Cl, on the formation of colored product FVS-NBD-Cl, [FVS] = 50 µg mL^−1^ at 55ºC for 10 min.

The most important factor affecting on the formation of yellow product was the concentration of NBD-Cl, (Fig. 3) shows that 0.75 mL of 0.2% w/v NBD-Cl solution gave maximum sensitivity. Increasing the volume of NBD-Cl leads to decrease in the absorbance; this may be due to the high background absorbance of the reagent.

**Figure 4:**
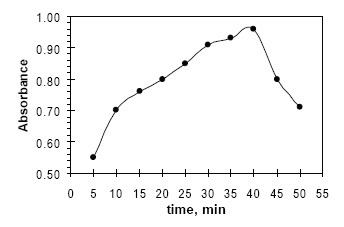
Effect of time of heating on the formation of colored product FVS-NBD-Cl, [FVS] = 50 µg mL^−1^ + 0.75 mL NBD-Cl 0.2% at 55ºC.

The influence of the time of heating was investigated in the rang of 5−50 min. The experimental results show that heating in the range 5–40 min gave the optimal values in kinetic studies (Fig. 4). The color product was stable for at least 2 days at room temperature.

### Quantitation methods

**Figure 5:**
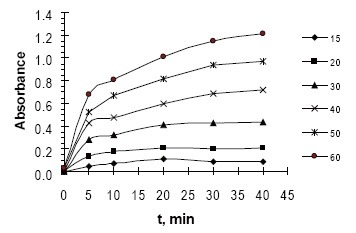
Absorbance-time curve for the reaction of FVS with NBD-Cl; [FVS] = 15–60 µg mL^−1^.

Because the intensity of the color increased with time (Fig. 5), this was used as the basis for a useful kinetic method for the determination of fluvastatin. The rate data, rate constant, variable time (fixed concentration or fixed absorbance) and fixed time methods (26, 27) were tested and the most suitable analytical methods were chosen regarding the applicability, sensitivity, the values of the intercept and correlation coefficient (R^2^).

**Figure 6.**
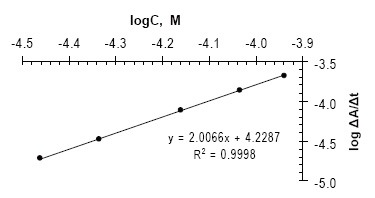
Calibration plot of logarithm rate of the reaction against logarithm molar concentration of FVS for rate data method.

**Rate data method.** The rate of reaction would follow a pseudo order rate constant and obeyed the following rate equation:

$$v=\Delta \text{A}/\Delta \text{t}={\text{k}}^{\prime }{\text{C}}^{\text{n}}$$

where ν is the reaction rate, A is the absorbance, t is the measuring time, k′ is the pseudo order rate constant, C is the concentration of the drug mol L^−1^ and n is the order of the reaction. A calibration curve was constructed by plotting the logarithm of the reaction rate (log ν) versus logarithm of drug concentration (log C) which showed a linear relationship over the concentration range of 15–50µg mL^−1^ (Fig. 6). The logarithmic form of the above equation is written as follows:

$$\text{Log}\;v=\text{Log}\;\Delta \text{A}/\Delta \text{t}=\text{Log}\;{\text{k}}^{\prime }+\text{n}\;\text{Log}\;\text{C}$$

Log v=Log ΔA/Δt=4.2287+2.0066 Log [FVS]

Thus, k′ = 16931.6 M^−1^ S^−1^, and the reaction is the second order (n=2.0066) with respect to FVS concentration. The limit of detection (LOD) and limit of quantification (LOQ) for rate data method were determined and were found to be 0.017 and 15 μg mL^−1^, respectively.

**Rate constant method.** Graphs of the reciprocal of absorbance versus time for FVS concentration in the range of 30−60 μg mL^−1^ (6.92 × 10^−5^ to 13.84 × 10^−5^, M) were plotted and all appeared to be rectilinear. Slopes are pseudo order rate constants (k′) corresponding to different FVS concentrations, and are presented in Table 1. Regression of C versus k′ gave the following equation:

$${\text{k}}^{\prime }=-0.00076+3.2943\text{C}\;\;\;\;\;({\text{R}}^{2}=0.9993)$$

**Table 1:** Values of rate constant K′

[FVS], M	k′(M^−1^S^−1^)

6.92 × 10^−5^	−5.3 × 10^−4^
9.23 × 10^−5^	−4.5 × 10^−4^
11.53 × 10^−5^	−3.8 × 10^−4^
13.84 × 10^–5^	−3.0 × 10^–4^

**Variable time method.** Reaction rate data were recorded for different FVS concentrations in the range 15−60 μg mL^−1^. A preselected value of the absorbance 0.68 was fixed and the time was measured in the seconds (Table 2). The reciprocal of time (1/t) versus the initial concentration of FVS was plotted and the following equation of calibration graph was obtained:

$$1/\text{t}=-0.0051+60.203\text{C}\;\;\;\;\;({\text{R}}^{2}=0.9868)$$

**Table 2:** Values of reciprocal time taken at fixed absorbance for the different rates of variable concentration of FVS at constant concentrations of NBD-Cl

[FVS], M	1/t (S^−1^)

9.23 × 10^−5^	5.56 × 10^−4^
11.53 × 10^−5^	16.67 × 10^−4^
13.84 × 10^−5^	33.33 × 10^−4^

The range of FVS concentrations giving the most satisfactory results was limited 40–60 μg mL^−1^ (9.23 × 10^−5^ to 13.84 × 10^−5^ M).

**Fixed time method.** At preselected fixed time, the absorbance of yellow colored solution containing varying amounts of FVS was measured at 55°C and 462 nm. Calibration graphs were constructed by plotting the absorbance against the initial concentration of FVS at fixed time 5, 10, 20, 30 and 40 min. The regression equations, correlation coefficients and linear ranges are given in (Table 3). Correlation coefficient, intercept and slope values for the calibration data calculated using the least squares method (28).

**Table 3:** Regression equations for FVS at fixed time and 55°C

Time (min)	Regression equation	Correlation coefficient	Linear range (μg mL^−1^)

5	A = 0.0137C − 0.1394	0.9963	15–60
10	A = 0.0162C − 0.1590	0.9990	15–80
20	A = 0.0200C − 0.1863	0.9998	10–90
30	A = 0.0227C − 0.2307	0.9968	10–90
40	A = 0.0233C − 0.2300	0.9957	10–90

A, Absorbance; C, Concentration.

It is clear that, the slope increases with time and the most acceptable values of the correlation coefficient, linear range and the intercept were obtained for a fixed time of 20 min. Therefore, the fixed time of 20 min. was utilized for the assay of FVS concentration. The limit of detection (LOD) and limit of quantification (LOQ) for fixed time (20 min) method were determined and were found to be 0.134 and 10 µg mL^−1^, respectively. For more accurate analysis, Ringbom optimum concentration range was calculated to be 15–50 μg mL^−1^. Table 4 shows the values of molar absorptivity, Sandell’s sensitivity and some analytical characteristics for fixed time (20 min) method.

**Table 4:** Analytical characteristics of the fixed time (20 min) method

Parameters	FVS	

λ_max_ (nm)	462	
Beer’s law limit (μg mL^−1^)	10–90	
Molar absorptivity (L mol^−1^ cm^−1^)	0.59 × 10^4^	
Stoichiometric relationship, FVS:NBD-Cl	1:1	1:2
Logarithmic formation constant	6.3	11.84
Optimum photometric range (μg mL^−1^)	15−50	
Detection limit (μg mL^−1^)	0.134	
Limit of quantification (μg mL^−1^)	10	
Sandell’s sensitivity (μg cm^−2^ per 0.001 absorbance unit)	0.147	
Regression equation^a^	A = 0.0200C − 0.1863	
Correlation coefficient, R^2^	0.9998	

a *A=mC+b*, where *C* is the concentration in μg mL^−1^ and *A* is the absorbance.

### Stoichiometric Relationship

**Figure 7:**
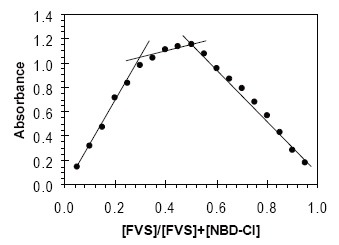
Job’s method of continuous variations; [FVS]+[NBD-Cl] =5.0 × 10^−4^ M

**Figure 8:**
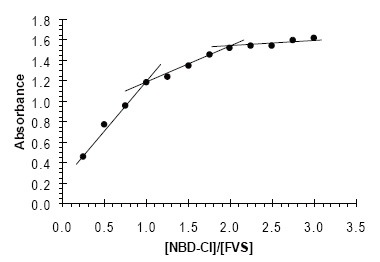
Mole-ratio method; [FVS] = 2 × 10^−4^ M, [NBD-Cl] = 5 × 10^−5^ – 6 × 10^−4^ M.

The composition of colored product was determined by Job’s method of continuous variation and mole-ratio method (29), for fixed time (20 min) method. It is apparent from the data that a molar ratio of 1:1 and 1:2 FVS to NBD-Cl (Fig. 7 and 8).

As result, the most acceptable values of the correlation coefficients were obtained for a rate data and fixed time (20 min) methods. Thus, they were used for the determination of FVS in pure form and pharmaceutical formulations.

### Analytical Methods Validation

The accuracy and precision of the proposed methods were carried out by six determinations at four different concentrations. Percentage relative standard deviation (RSD%) as precision and percentage relative error (Er%) as accuracy of the suggested methods were calculated. Table 5 shows the values of relative standard deviations for different concentrations of the FVS determined from the calibration curves. These results of accuracy and precision show that the proposed methods have good repeatability and reproducibility. The proposed methods were found to be selective for the estimation of FVS in the presence of various capsule excipients. For this purpose, a powder blend using typical capsule excipients was prepared along with the drug and then analyzed. The recoveries were not affected by the excipients and the excipients blend did not show any absorption in the range of analysis.

**Table 5:** Accuracy and precision for the determination of FVS in bulk powder by the proposed methods (rate data and fixed time)

Method	FVS, μg mL^−1^		Er%	RSD (%)	%Recovery ± S.D.
	Taken	Found a			

Rate data	20.00	20.28	1.41	2.38	101.41±0.48
	30.00	30.27	0.91	1.93	100.91±0.59
	40.00	40.20	0.49	1.54	100.49±.0.62
	50.00	50.23	0.46	1.01	100.46±0.51
Fixed time	10.00	10.08	0.80	2.27	100.80±0.23
	30.00	30.24	0.80	1.56	100.80±0.47
	50.00	50.62	1.25	1.11	101.25±0.56
	70.00	70.22	0.32	0.70	100.32±0.49

a Average of six determinations.

### Application to the pharmaceutical dosage forms

The performance of the proposed methods was assessed by comparison with the official non-aqueous titration method for FVS (30). Mean values were obtained with a Student’s *t*- and *F*-tests at 95% confidence limits for four degrees of freedom. The results showed comparable accuracy (*t*-test) and precision (*F*-test), since the calculated values of *t*- and *F*-tests were less than the theoretical data.

The proposed procedures were applied to determine FVS in its pharmaceutical formulations. The results in Table 6 indicate the high accuracy and precision. As can be seen from Table 6, the proposed methods have the advantages of being virtually free from interferences by excipients such as glucose, lactose, and starch or from common degradation products. The results obtained were compared statistically by the student’s *t*-test (for accuracy) and the variance ratio *F*-test (for precision) with those obtained by the official method for the samples of the same batch (Table 6). The values of *t*- and *F*-tests obtained at 95% confidence level did not exceed the theoretical tabulated value indicating no significant difference between the methods compared.

**Table 6:** Application of the proposed methods to the determination of FVS in dosage forms

Sample	%Recovery^a^ ± S.D.		
	Proposed methods		Official method
	Rate data	Fixed time	

Pure FVS	100.82 ± 0.55	100.79 ± 0.44	99.60 ± 0.28
*t*-value	1.02	1.36	
*F*-value	3.85	2.47	
Almastatin capsules (20mg)			
*X* ± S.D.^a^	100.48 ± 0.18	100.16 ± 0.13	100.64 ± 0.11
*t*-value^b^	2.40	1.35	2.13
*F*-value^b^	2.68	1.40	
Almastatin capsules (40mg)			
*X* ± S.D.^a^	101.17 ± 0.23	99.99 ± 0.37	99.84 ± 0.17
*t*-value^b^	2.32	1.07	1.92
*F*-value^b^	1.83	4.74	
Fluvastatinvcapsules (20mg)			
*X* ± S.D.^a^	100.37 ± 0.31	100.75 ± 0.42	100.28 ± 0.23
*t*-value^b^	1.05	2.02	2.06
*F*-value^b^	1.81	3.33	
Fluvastatinvcapsules (40mg)			
*X* ± S.D.^a^	100.53 ± 0.21	100.13 ± 0.20	101.05 ± 0.09
*t*-value^b^	1.11	1.05	1.78
*F*-value^b^	5.44	4.94	

a Five independent analyses;

b Theoretical values for *t* and *F*-values at five degree of freedom and 95% confidence limit are (*t* =2.776) and (*F*=6.26).

### Mechanism of the Color Reaction

**Figure 9:**
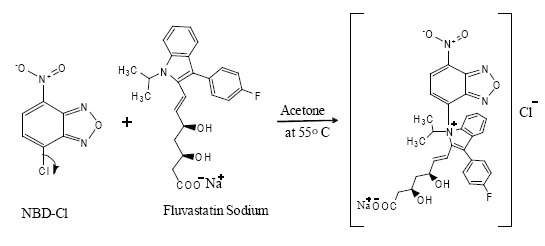
The proposed pathway of the reaction between FVS and NBD-Cl.

A nitro group in NBD-Cl formula reduces the ring activity, especially at para position, so the nitrogen in FVS bonds to this position forming a colored product. Therefore, NBD-Cl formula loses an anion chloride and the ring returns to its aromaticity. The reaction mechanism is shown in Fig. 9.

## CONCLUSION

The proposed kinetic spectrophotometric method is the first kinetic method for the determination of fluvastatin. It is selective, reproducible, accurate and precise and hence can be used for the routine quality control of fluvastatin in bulk and pharmaceutical formulations. 4-chloro-7-nitrobenzofurazan (NBD-Cl) was used as reagent in acetone medium. The sample recoveries from all formulations were in good agreement with their respective label claims, which suggested non-interference of formulations excipients in the estimation.
